# An Intra‐Interpersonal Vulnerability Model for Understanding Trauma Conditions: Factors in the Development and Maintenance of PTSD and Complex PTSD

**DOI:** 10.1002/cpp.70333

**Published:** 2026-09-01

**Authors:** Reuben Kindred, Maja Nedeljkovic, Glen Bates

**Affiliations:** ^1^ Department of Psychological Sciences Swinburne University of Technology Melbourne Australia

**Keywords:** attachment insecurity, complex PTSD, emotion regulation, PTSD, social connectedness, trauma

## Abstract

Adverse childhood events and insecure attachment lead to difficulties in emotion regulation and social support, predisposing individuals to post‐traumatic stress disorder (PTSD) and complex PTSD (CPTSD) when exposed to potentially traumatic events. However, the field lacks an integrated account of how these risk and protective factors combine, including the pathways through which attachment‐related vulnerability contributes to trauma symptoms. This study tested a proposed intra‐interpersonal vulnerability model using structural equation modelling with a sample of 706 participants (*M* = 30.64 years, SD = 11.00). Model fit was strong for symptoms of ICD‐11 PTSD (CFI = 0.969; SRMR = 0.055) and ICD‐11 CPTSD (CFI = 0.976; SRMR = 0.056). The model explained 35% of variance in PTSD symptoms and 75.7% of variance in CPTSD symptoms. Cross‐validation supported the generalisability of the model, with the overall pattern of effects also replicated among participants with elevated PTSD/CPTSD symptoms. As expected, adverse childhood events were linked to attachment insecurity (e.g., *β* = 0.29), which influenced emotional identification, emotion dysregulation and social connectedness (*β* = 0.24 to *β* = −0.69). Contrary to expectations attachment insecurity did not impact either PTSD or CPTSD trauma symptoms directly. Emotion dysregulation was significantly associated with both PTSD and CPTSD symptoms, whereas social connectedness was associated with CPTSD but not PTSD symptoms. Overall, the results indicate that risk and protective factors build upon each other, reinforcing the importance of developmental aspects in the emergence of post‐traumatic stress. Limitations include a community sample and cross‐sectional data, suggesting a need for longitudinal research.

## Summary

- Trauma symptoms may emerge through interacting intrapersonal and interpersonal vulnerabilities, including attachment insecurity, emotion dysregulation and social connectedness.
- Emotion identification and regulation may represent clinically modifiable mechanisms underlying PTSD and CPTSD symptoms.
- Attachment insecurity appears to influence trauma outcomes indirectly through socio‐affective processes rather than directly.
- Interventions targeting emotional clarity and regulation may be relevant across PTSD and CPTSD, while social connectedness may be particularly relevant to CPTSD symptoms.

## 1 Introduction

Post‐traumatic stress disorder (PTSD) and complex PTSD (CPTSD) are distinct trauma‐related disorders within the International Classification of Diseases (ICD‐11; World Health Organization 2019). ICD‐11 PTSD comprises three symptom clusters: re‐experiencing, avoidance and a persistent sense of current threat. CPTSD—which is commonly developed after exposure to prolonged or repeated trauma—includes these core PTSD symptoms alongside disturbances in self‐organisation (DSO), comprising affective dysregulation, negative self‐concept and interpersonal difficulties (for a review, see Kindred et al. 2026). Although trauma exposure is common (Kessler et al. 2017), lifetime PTSD prevalence is approximately 5.6% (Koenen et al. 2017) and CPTSD point prevalence is 6.2% (Huynh et al. 2025). Thus, trauma exposure alone is insufficient to explain why some individuals develop PTSD or CPTSD while others do not, highlighting the importance of understanding developmental, emotional and interpersonal vulnerabilities that may contribute to post‐traumatic stress outcomes. However, these factors have typically examined in isolation, and the field lacks an integrated account of how they combine to shape PTSD and CPTSD outcomes. This study proposes an intra‐interpersonal vulnerability model, exploring how attachment insecurity, emotion identification, regulation and social connectedness predispose individuals to post‐traumatic stress outcomes. As adverse childhood experiences (ACEs) represent both exposure and developmental risk, attachment is discussed first as a central vulnerability process shaping later emotional and interpersonal functioning.

### 1.1 Attachment and Trauma

Attachment can be conceptualised as relatively enduring patterns of expectations, emotions and behaviours that shape how individuals seek security and support within close relationships (Mikulincer and Shaver 2016). Attachment plays a pertinent role within traumatic conditions due to its impact on the biological stress system. In adulthood, insecure attachment is expressed in stress‐response strategies under threat, such as hyperactivation (heightened monitoring and distress amplification) and deactivation (emotional suppression and interpersonal distancing) (Shaver and Mikulincer 2014). These, in turn, can impair access to social resources and successful emotional regulation (Sroufe et al. 1999). Adult attachment styles reflect enduring relational expectations shaped by caregiving and later relational experiences and influence responses to stress and support seeking (Fraley and Shaver 2000; Opie et al. 2021).

While the present study assessed adult attachment, not childhood attachment, adult attachment patterns are widely understood to reflect theoretically meaningful, though indirect, markers of developmental risk (Mikulincer and Shaver 2016), these are particularly salient in the context of stressful or traumatic periods, which often occur in early adulthood (Kessler et al. 2017). Attachment patterns show both stability and change across development (Opie et al. 2021) and empirical studies have linked ACEs to insecure adult attachment styles (Riggs and Kaminski 2010). For instance, a large prospective cohort analysis (Papalia and Widom 2024) found that childhood maltreatment and neglect—conceptually overlapping with the relational core of the ACE framework—predicted adult attachment insecurity. Early abusive and inconsistent caregiving is proposed to disrupt the development of secure internal working models and thus foster enduring attachment insecurity across the lifespan (Riggs and Kaminski 2010).

In turn, insecure attachment has been associated with greater PTSD symptom severity following trauma exposure (Ayers et al. 2014; Franz et al. 2014; London et al. 2015; Ortigo et al. 2013), highlighting the role of socio‐affective vulnerability. However, the mechanisms from attachment styles and patterns to trauma outcomes are not comprehensively understood. Based on this literature, attachment insecurity would contribute to trauma‐related symptoms, either directly or indirectly via transdiagnostic mechanisms. Existing research suggests multiple mediating pathways, including emotion regulation, cognitive appraisals, self‐esteem and social support (Harris et al. 2024; Lim et al. 2020).

### 1.2 Intrapersonal Factors and Trauma

Difficulties in emotion identification, or recognising one's own emotional processes, may undermine broader regulatory functioning, thereby increasing vulnerability to psychological disorders. Emotional clarity enables recognition of one's emotions which enables the resolution of discrepancies between current and desired emotions (Putica et al. 2022). Ehring and Quack (2010) found that emotional clarity remained uniquely associated with early interpersonal trauma after accounting for PTSD severity. Difficulties identifying feelings, a facet of alexithymia, may also contribute to PTSD through impaired emotion regulation. A meta‐analysis of 43 samples found difficulties in identifying feelings were more strongly associated with PTSD than other alexithymia subdimensions and closely linked to emotion dysregulation (Edwards 2022), suggesting it may impair trauma processing indirectly via deficits in emotional regulation (e.g., emotional processing theory; Rauch and Foa 2006).

Beyond emotion identification, difficulties with regulating emotions more broadly are also linked to the development and maintenance of PTSD symptoms (Bardeen et al. 2013; Ehlers and Clark 2000; Kleim et al. 2007). This is unsurprising, as difficulties in emotion regulation are strongly implicated in PTSD and CPTSD and individuals with PTSD have disruptions in affective information processing (Ford 2015). General emotion regulation abilities (Gratz and Roemer 2004) have been consistently associated with PTSD (Ehring and Quack 2010; Seligowski et al. 2015; Weiss et al. 2012) and CPTSD (Cloitre et al. 2021; Gelezelyte et al. 2022). In a longitudinal study, Bardeen et al. (2013) found that pre‐existing emotion regulation difficulties predicted higher post‐traumatic stress symptoms immediately and 8 months after a campus shooting. These findings support conceptualising emotion dysregulation as vulnerability factor in trauma responses. Consistent with Bardeen et al. (2013), we operationalised emotion dysregulation in terms of reduced regulatory abilities under distress (e.g., non‐acceptance, difficulties pursuing goals, impulse control difficulties and limited access to effective strategies), while modelling emotion identification separately as an upstream process.

Beliefs about emotional controllability may represent another vulnerability factor in PTSD and related conditions. O'Brien et al. (2025) identified beliefs about emotional uncontrollability as contributing to emotional disengagement and trauma severity. Individuals who perceive emotions as controllable are more likely to use adaptive strategies such as cognitive reappraisal, whereas those who view emotions as uncontrollable tend to rely on suppression, exacerbating distress (Kneeland et al. 2016). However, it remains unclear whether these beliefs operate similarly across PTSD and CPTSD, as direct comparisons are limited. The present study extends this work by testing controllability beliefs within a model that includes CPTSD.

### 1.3 Interpersonal Factors and Trauma

Social connectedness has been proposed as another explanatory mechanism underlying the association between insecure attachment and post‐traumatic stress (Lim et al. 2020). Social connectedness can be critical for people affected by trauma and the evidence indicates that social processes should be viewed as an essential part of biological and cognitive models (Bryant 2023). Consistent with this, recent cascade models of CPTSD emphasise how childhood trauma and attachment insecurity may influence trauma outcomes through socio‐interpersonal pathways, including social support, acknowledgment and relational functioning (e.g., Maercker et al. 2022). In this study, we treated social connectedness as comprising both social support and subjective connection.

Social support refers to the perceived availability of emotional and practical assistance from others (Cohen and Wills 1985). Supportive relationships may be critical after trauma because they can buffer threat responses, facilitate emotion regulation and promote recovery through emotional and practical assistance (Bryant 2023). A meta‐analysis of longitudinal studies by Wang et al. (2021) showed that social support had a robust protective effect on PTSD over time, consistent across symptom levels, sample types and age, highlighting its robustness as both a protective and maintaining factor.

In addition to social support being linked to lower levels of PTSD symptomatology, subjective experiences of connection and disconnection, such as loneliness, may also be central in trauma recovery frameworks (e.g., Hobfoll 1989). We focused on emotional loneliness because it captures perceived deficits in closeness and emotional bonding and has been linked to PTSD symptom trajectories (Heinrich and Gullone 2006). Individuals may have support available yet still feel emotionally disconnected, particularly under distress. Longitudinal evidence from Fox et al. (2021) demonstrates that both emotional and social loneliness predicted increases in PTSD symptoms over time, while elevated PTSD symptoms also predicted subsequent increases in loneliness. This cyclical dynamic suggests that loneliness both contributes to and is exacerbated by trauma‐related distress, highlighting the importance of addressing social connection as part of trauma recovery interventions.

### 1.4 The Present Study

Together, the literature suggests that post‐traumatic stress outcomes emerge through the interplay of attachment‐related vulnerabilities, emotion regulation impairments and disrupted social connectedness. These findings collectively support a multilevel model that integrates both intrapersonal and interpersonal mechanisms.

This study's intra‐interpersonal vulnerability model draws on adult attachment theory (Fraley and Shaver 2000), transtheoretical models of emotion dysregulation (e.g., Gratz and Roemer 2004; Sheppes et al. 2015) and social causation theory (Dohrenwend 2000). Although ACEs represent the distal adversity variable examined in the model, the model does not assume that current PTSD or CPTSD symptoms necessarily arose from these childhood experiences or exclude the contribution of traumatic experiences later in life. We expected the same pattern of associations for both ICD‐11 PTSD and CPTSD outcomes. We hypothesised that (1) ACEs would be associated with trauma symptoms both directly and indirectly through their influence on attachment insecurity and emotion regulation capabilities; (2) insecure attachment would show a total association with trauma symptoms potentially through both direct and indirect pathways. As a separate research question, we examined attachment anxiety and attachment avoidance separately to explore whether they show distinct pathways to trauma symptoms; (3) emotion identification would mediate the relationship between attachment insecurity and emotion regulation difficulties, which in turn would contribute to trauma symptoms; and (4) social connectedness would mediate the relationship between attachment insecurity and trauma symptoms.

## 2 Method

### 2.1 Participants and Procedure

The final sample consisted of 706 English‐speaking adults who had experienced a Diagnostic and Statistical Manual of Mental Disorders (5th ed., text rev.; DSM‐5‐TR; American Psychiatric Association 2022) Criterion A trauma (e.g., sexual assault, natural disasters and witnessing a violent death).

Participants were recruited using nonprobability sampling, with recruitment targeted towards trauma‐exposed adults. Advertisements were distributed through social media, trauma‐ and mental health–focused online groups and forums and a university‐based research participation system. Recruitment materials described the study as examining psychological responses to traumatic experiences. Participants reported their traumatic event or events during the survey, which were screened for consistency with DSM‐5‐TR Criterion A. Those for whom a qualifying traumatic event could not be established were excluded, and the ITQ was completed with reference to the nominated traumatic event.

Participants were excluded if they completed less than 95% of all survey items, reflecting a prespecified conservative data‐completeness criterion. Participants were also excluded if they correctly answered fewer than two of three attention checks, consistent with recommendations for identifying inattentive responding (Abbey and Meloy 2017), completed the survey in under 10 min or did not report an event consistent with the trauma exposure requirement. Following Kline's (2015) conservative guideline of 20 participants per estimated parameter, the most complex model with 35 parameters would require a minimum of 700 participants. With 706 participants, this criterion was met, with a ratio of 1:20.17.

The survey took a median of 27 min to complete. Participants accessed the survey via a website link and were entered into a prize draw for Amazon gift cards. Data were collected online between August 2023 and June 2024. Ethical approval was obtained from an institutional Human Research Ethics Committee.

### 2.2 Measures

#### 2.2.1 ACEs

The Revised Adverse Childhood Experiences Questionnaire (Finkelhor et al. 2015) is a 14‐item binary scale (yes/no) measure used to assess exposure to adverse experiences during childhood, which includes exposure to physical abuse, sexual abuse, neglect and household dysfunction. The ACE questionnaire demonstrated good internal consistency (*α* = 0.80) in the current sample. The average number of ACEs was 4.89 (SD = 3.3).

#### 2.2.2 PTSD and CPTSD Symptomatology

The International Trauma Questionnaire (ITQ; Cloitre et al. 2018) is an 18‐item self‐report measure assessing ICD‐11 PTSD and CPTSD. It comprises two subscales, PTSD (including re‐experiencing, avoidance and sense of threat) and DSO (including affective dysregulation, negative self‐concept and relational disturbance). Items are rated from 0 (*not at all*) to 4 (*extremely*). Higher scores reflect greater PTSD and CPTSD symptom severity. The ITQ demonstrated excellent internal consistency: total score *α* = 0.93, PTSD *α* = 0.88 and DSO *α* = 0.92.

#### 2.2.3 Attachment

The Experiences in Close Relationships—Short Form (ECR‐SF; Wei et al. 2007) is a 12‐item self‐report questionnaire that measures adult attachment styles. It includes two subscales—attachment anxiety and attachment avoidance—each consisting of six items. Responses are rated on a 7‐point Likert scale ranging from 1 (*strongly disagree*) to 7 (*strongly agree*). The ECR‐SF has demonstrated good psychometric properties, including strong internal consistency and test–retest reliability (Wei et al. 2007). In this sample, Cronbach's alpha was *α* = 0.77 overall, *α* = 0.78 for attachment anxiety and *α* = 0.83 for attachment avoidance.

In the current study, attachment insecurity was operationalised as a summed score rather than a latent construct due to low factor loadings for anxiety (0.47) and avoidance (0.43). Prior research supports this approach, indicating that a combined score reliably captures relational vulnerability and predicts emotional outcomes (Fraley et al. 2011; Wei et al. 2007).

### 2.3 Mediating Variables

#### 2.3.1 Emotion Identification

Given that emotion identification is a multifaceted construct involving both the awareness (Lane and Schwartz 1987) and differentiation of emotions (Barrett et al. 2001; Barrett 2016), we utilised three established subscales from different measures to provide a more comprehensive assessment. These included the Emotional Clarity subscale of the Trait Meta‐Mood Scale (TMMS; Salovey et al. 1995; eight items, *α* = 0.91), the Difficulty Identifying Feelings subscale of the TAS (Bagby et al. 1994; seven items, *α* = 0.89) and the *Clarity* subscale of the Difficulties in Emotion Regulation Scale (DERS) (Gratz and Roemer 2004; five items, *α* = 0.91). All items were rated on 5‐point Likert scales, and each subscale demonstrated strong internal consistency in the current sample. These subscales were selected to provide complementary coverage of emotion identification rather than relying on a single facet. Although all three assess clarity or identification of emotional experience, they differ in theoretical emphasis and item content, reflecting trait‐level emotional insight (TMMS), deficit‐oriented difficulty identifying feelings (TAS) and emotional clarity under emotional distress (DERS). Using multiple indicators from distinct measures improves construct validity by modelling variance common to emotion identification while attenuating scale‐specific method variance (Podsakoff et al. 2003). This multi‐indicator approach may also better approximate emotion identification in trauma‐exposed populations, where emotional insight is often state dependent and may not be fully captured by a single self‐report scale (Ford 2015).

#### 2.3.2 Emotion Regulation

The DERS (Gratz and Roemer 2004) is a 36‐item self‐report questionnaire that looks at an individual's difficulties in regulating their emotions, including lack of emotional awareness, non‐acceptance of emotional responses, difficulties engaging in goal‐directed behaviour, impulse control difficulties and limited access to emotion regulation strategies. Items are rated on a 5‐point Likert scale. In the current study, the DERS showed excellent internal consistency (*α* = 0.95). Excluding the Clarity subscale, reliability remained strong (*α* = 0.94). Subscale alphas were Non‐Acceptance (*α* = 0.93), Goals (*α* = 0.88), Impulse (*α* = 0.91), Awareness (*α* = 0.87) and Strategies (*α* = 0.91).

Personal beliefs about the controllability of emotions were measured using the four‐item Personal Beliefs About Emotions Scale (De Castella et al. 2013), which assesses the extent to which individuals believe in their ability to control or change their emotions. Participants rated their agreement with each statement on a 5‐point Likert scale. The scale demonstrated good reliability in the current study, with a Cronbach's alpha of *α* = 0.83.

#### 2.3.3 Social Connectedness

Perceived social support was assessed using the 12‐item Multidimensional Scale of Perceived Social Support (MSPSS; Zimet et al. 1988), which measures support from family, friends and significant other. Items are rated on a 7‐point Likert scale, with higher scores indicating greater perception of social support. Internal consistency was excellent (*α* = 0.93) in the current study.

Emotional loneliness was measured using the six‐item Emotional Loneliness subscale of the de Jong Gierveld Loneliness Scale (DJGLS; de Jong Gierveld and Van Tilburg 2006), assessing the absence of close relationships. Emotional loneliness reflects a core experiential component of social connectedness and captures perceived deficits in emotional bonding rather than the objective size of one's social network (de Jong Gierveld and Kamphuis 1985). Items are rated on a 5‐point Likert scale, and in the current study, internal consistency was good, with a Cronbach's alpha of *α* = 0.87.

### 2.4 Data Analysis

All analyses were conducted with version 4.0.3 of R (R Core Team 2018), using the lavaan (Rosseel 2011), mice (van Buuren and Groothuis‐Oudshoorn 2011), sem (Fox 2006), cvsem (Huang 2020) and car (Fox and Weisberg 2019) packages.

Missing data (0.16%) were handled using multiple imputation with predictive mean matching. Little's MCAR test was significant (*χ* ^2^(23, 273) = 23,755.50, *p* = 0.013); however, *t*‐tests showed no significant differences on key variables, which supports the assumption of data being missing at random. Skewness (≤ 0.87) and kurtosis (≥ −1.25) values were within acceptable ranges, which indicates normality (Hair et al. 2010).

**FIGURE 1:**
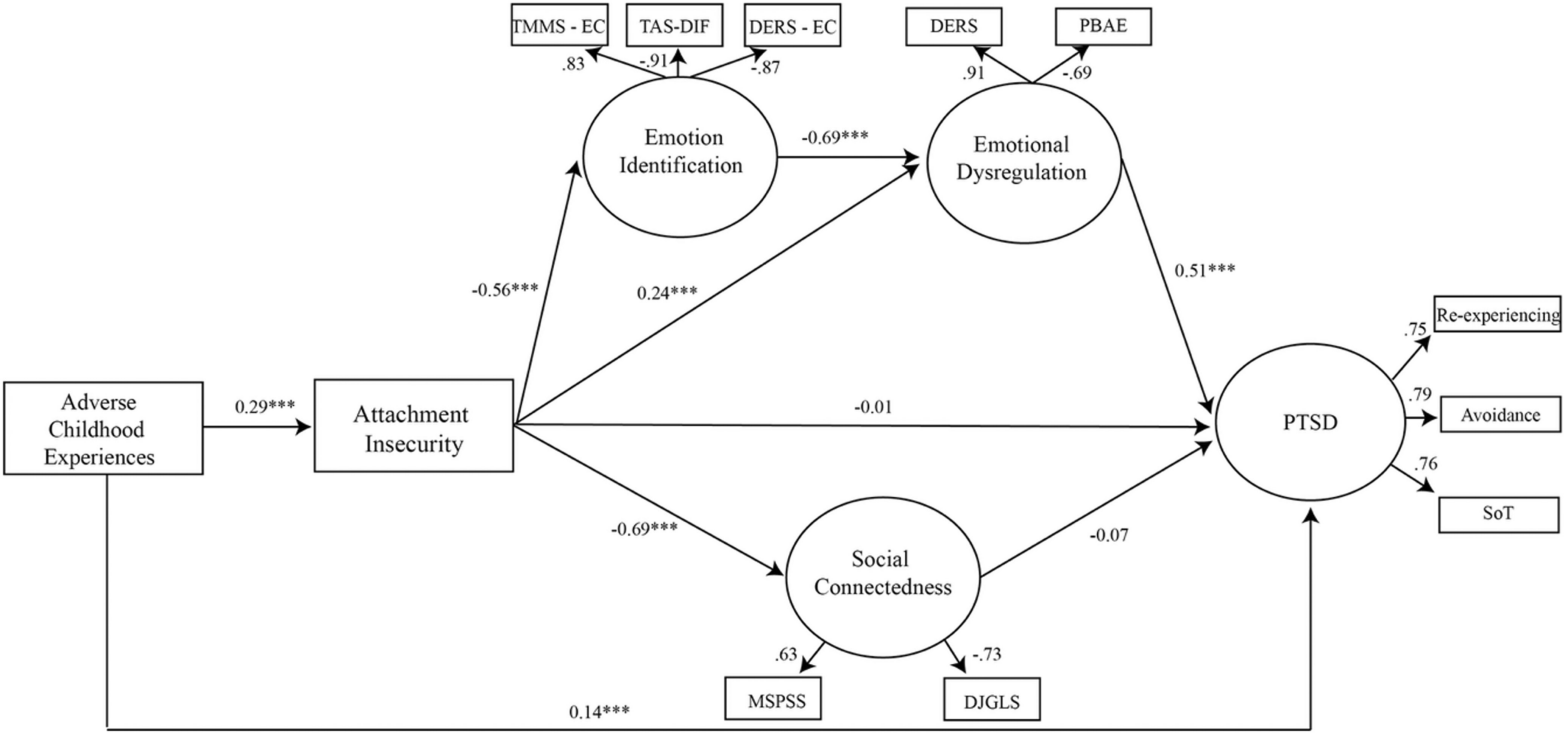
Structural equation model for ICD‐11 PTSD symptoms. Standardised *β* coefficients are displayed. Residual covariances between emotion identification and social connectedness, and emotional dysregulation and social connectedness, are omitted for clarity. *p* < 0.001. DERS‐EC, Difficulties in Emotion Regulation Scale—Emotional Clarity; DJGLS, de Jong Gierveld Loneliness Scale; MSPSS, Multidimensional Scale of Perceived Social Support; SoT, sense of threat; TAS‐DIF, Toronto Alexithymia Scale—Difficulty Identifying Feelings; TMMS‐EC, Trait Meta‐Mood Scale—Emotional Clarity.

**FIGURE 2:**
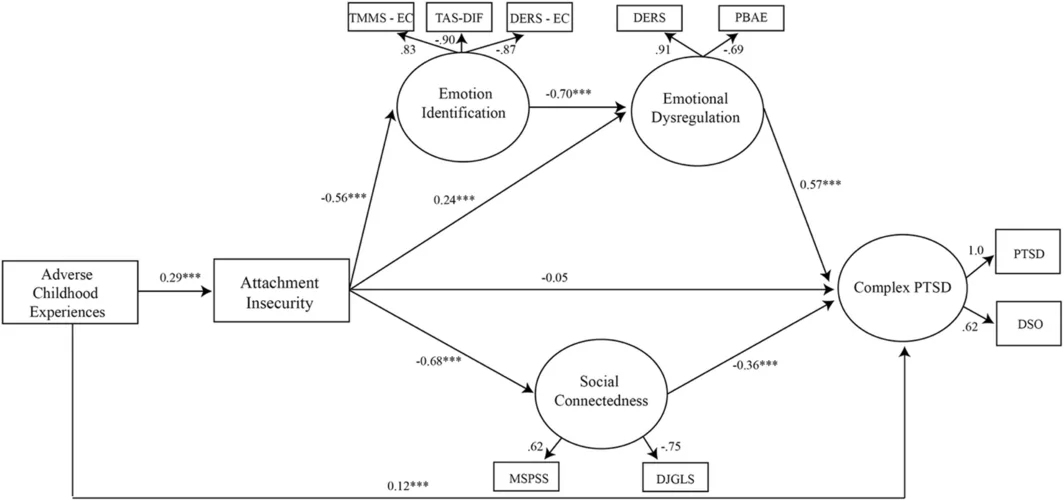
Structural equation model for ICD‐11 CPTSD symptoms. Standardised *β* coefficients are displayed. Residual covariances between emotion identification and social connectedness, and emotional dysregulation and social connectedness, are omitted for clarity. *p* < 0.001. DERS‐EC, Difficulties in Emotion Regulation Scale—Emotional Clarity; DJGLS, de Jong Gierveld Loneliness Scale; DSO, disturbances in self‐organisation; MSPSS, Multidimensional Scale of Perceived Social Support; TAS‐DIF, Toronto Alexithymia Scale—Difficulty Identifying Feelings; TMMS‐EC, Trait Meta‐Mood Scale—Emotional Clarity.

Structural equation modelling (SEM) was conducted using maximum likelihood estimation with 10,000 bootstrapped samples to generate 95% bias‐corrected confidence intervals for direct, indirect and total effects. Model fit was evaluated using RMSEA (< 0.08 adequate, < 0.06 excellent), CFI and TLI (> 0.90 adequate, > 0.95 excellent) and SRMR (≤ 0.08 acceptable, ≤ 0.05 good), following established guidelines (Bentler 1990; Hu and Bentler 1999; Steiger 1990). To reduce overfitting, model modifications were limited to three per model, with only indices ≥ 20 in *χ* ^2^ units considered (MacCallum 1986; Byrne 2010). For model identification, residual covariances were estimated between emotion identification and social connectedness and between emotional dysregulation and social connectedness (not depicted in Figures 1 and 2 for clarity).

*k*‐fold cross‐validation was used to assess model generalisability. The data were split into five equal subsets, with the model trained on *k* − 1 folds and validated on the remaining fold. Performance of the model was averaged across all iterations. This provides a more robust estimate of predictive accuracy than a single train‐test split, which utilises only a portion (e.g., a 20% testing set) of the sample for validation.

Sensitivity analyses re‐estimated the PTSD and CPTSD models among participants meeting ICD‐11 symptom‐cluster thresholds for either PTSD or CPTSD. The CPTSD model was additionally re‐estimated controlling for trauma repetition. Exploratory analyses examined gender differences using Welch's independent‐samples *t*‐tests and associations between attachment dimensions and the six ICD‐11 PTSD and DSO symptom clusters using Pearson correlations, with false discovery rate (FDR) correction applied for multiple comparisons.

## 3 Results

A total of 1198 individuals participated in the survey. Thirteen multivariate outliers (Mahalanobis distance > 56.893) were removed. After applying data integrity criteria, the final sample consisted of 706 participants.

### 3.1 Descriptive Statistics

Participants (*N* = 706) ranged from 18 to 69 years (M = 30.64, SD = 11.00), with most identifying as female (*n* = 542, 76.8%). ICD‐11 diagnoses were indicated in 33.85% for PTSD and 23.08% for CPTSD. Common trauma types included sexual trauma (20.82%), domestic violence (14.73%) and medical/injury‐related trauma (7.93%). Overall, 45.5% reported having received a psychiatric diagnosis, 47.3% received psychiatric treatment, 12.18% reported receiving a formal PTSD diagnosis and 8.22% reported receiving a CPTSD diagnosis. Table 1 presents full demographic details.

**TABLE 1:** Demographic information about sample.

Category	*N*	Percentage (%)
Gender		
Male	132	18.70%
Female	542	76.80%
Non‐binary	23	3.30%
Other	7	1.00%
Nationality		
Australian	551	78.00%
United States	87	12.30%
Non‐UK Europe	21	3.00%
UK	15	2.10%
Canada	10	1.40%
Asia	6	0.90%
Africa	2	0.30%
Non‐Australian Oceania	2	0.30%
South America	1	0.10%
Ethnicity		
Unsure/prefer not to say	11	1.60%
Asian	68	9.60%
Black/African	12	1.70%
Hispanic/Latino	17	2.40%
Indian	35	5.00%
Indigenous	8	1.10%
Middle Eastern	19	2.70%
Multiracial	29	4.10%
White/Caucasian	468	66.30%
Other	36	5.10%
Employment		
Not employed	146	20.70%
Casual	137	19.40%
Part time	158	22.40%
Full time	262	37.10%
Education		
Secondary (i.e., Year 10)	9	1.30%
Secondary (i.e., Year 12)	272	38.50%
Vocational/technical school	171	24.20%
Postgraduate degree	68	9.60%
Bachelor's degree	156	22.10%
Unsure/prefer not to say	28	4.00%
Relationship status		
Single, never in a relationship	68	9.60%
Single	193	27.30%
In a relationship	198	28.00%
Married/de facto	210	29.70%
Divorced	32	4.50%
Widow	2	0.30%
Parental status		
No children	480	68.00%
Yes	224	31.70%

Exploratory analyses were conducted to examine gender differences across the study variables (Table S1). Females reported statistically significantly higher ACEs (FDR‐adjusted *p* = 0.016, *d* = 0.29) and emotion regulation difficulties (FDR‐adjusted *p* = 0.016, *d* = 0.28) than males. The effect sizes for both differences were small. No other gender differences remained statistically significant following correction.

### 3.2 Multicollinearity and Bivariate Correlations

Multicollinearity diagnostics showed VIF values between 1.02 and 1.48 and tolerance values between 0.68 and 0.98, which suggests no multicollinearity concerns. Most study variables were significantly correlated at *p* < 0.001, except for the modest correlation between attachment anxiety and avoidance (*r* = 0.12, *p* < 0.01). The number of significant associations suggests constructs are meaningfully related and the hypothesised pathways in the structural equation models are plausible. Table 2 presents means, standard deviations, skew, kurtosis and bivariate correlation analyses for the study variables.

**TABLE 2:** Mean, standard deviation, normality and bivariate correlations between study variables.

Variables	Range	*M*	SD	Skew	Kurtosis	1.	2.	3.	4.	5.	6.	7.	8.	9.	10.	11.	12.
1. ACEs	0–14	4.88	3.30	0.29	−0.66	—											
2. Att_anxiety	6–42	23.42	8.02	−0.10	−0.67	0.25	—										
3. Att_avoidance	6–42	14.41	8.20	0.22	−0.55	0.18	0.12	—									
4. Att_total	12–84	37.83	12.18	−0.11	−0.29	0.29	0.74	0.76	—								
5. TMMS	11–55	33.67	9.44	0.04	−0.51	−0.27	−0.27	−0.43	−0.47	—							
6. TAS‐DIF	7–35	19.69	7.33	−0.04	−1.01	0.35	0.35	0.41	0.51	−0.75	—						
7. DERS Clarity	5–25	12.76	4.80	0.42	−0.57	0.26	0.26	0.44	0.47	−0.86	0.79	—					
8. DERS Nonclarity	31–155	88.67	23.65	0.08	−0.59	0.28	0.46	0.39	0.57	−0.63	0.67	0.66	—				
9. PBAE	4–20	13.33	3.51	−0.31	−0.40	−0.18	−0.37	−0.25	−0.41	0.49	−0.51	−0.49	−0.63	—			
10. de Jong	6–30	18.20	5.80	−0.07	−0.73	0.23	0.42	0.29	0.47	−0.42	0.46	0.42	0.51	−0.41	—		
11. MSPSS	12–84	56.74	16.64	−0.41	−0.26	−0.36	−0.20	−0.50	−0.47	0.36	−0.35	−0.36	−0.39	0.30	−0.46	—	
12. ITQ total	0–48	21.08	11.41	0.20	−0.87	0.37	0.38	0.38	0.51	−0.50	0.61	0.53	0.68	−0.50	0.50	−0.40	—

*Note:* *N* = 706. All correlations are Pearson's *r*. All correlations are *p* < 0.001, except for the correlation between attachment anxiety and avoidance at *p* < 0.01.

Abbreviations: ACEs, adverse childhood experiences; Att_anxiety, attachment anxiety; Att_avoidance, attachment avoidance; Att_total, combined attachment score; de Jong, de Jong Gierveld Loneliness Scale; DERS Clarity, Difficulties in Emotion Regulation Scale—Clarity subscale; DERS Nonclarity, Difficulties in Emotion Regulation Scale—Non‐Acceptance, Goals, Impulses, Awareness, Strategies; ITQ total, ITQ full score; MSPSS, Multidimensional Scale of Perceived Social Support; PBAE, positive beliefs about emotions; TAS‐DIF, Toronto Alexithymia Scale—Difficulty Identifying Feelings subscale; TMMS, Trait Meta‐Mood Scale.

### 3.3 Measurement Model

Using the established two‐step approach (Anderson and Gerbing 1988), a confirmatory factor analysis was conducted to first estimate the measurement model. The PTSD measurement model showed acceptable fit: *χ* ^2^(41) = 180.15, *p* < 0.001, CFI = 0.969, TLI = 0.950, RMSEA = 0.069 and SRMR = 0.042. All factor loadings were statistically significant and ≥ 0.66. The CPTSD measurement model demonstrated acceptable‐to‐good fit to the data: *χ* ^2^(34) = 124.546, *p* < 0.001, CFI = 0.980, TLI = 0.967, RMSEA = 0.061 and SRMR = 0.035. All factor loadings were statistically significant and ≥ 0.62.

### 3.4 Structural Equation Model

#### 3.4.1 Post‐Traumatic Stress Symptoms

After establishing an acceptable fitting measurement model for ICD‐11 PTSD symptoms, we fit the structural model. The PTSD SEM model showed good fit, *χ* ^2^(44) = 181.29, *p* < 0.001, CFI = 0.969, TLI = 0.954, RMSEA = 0.066 and SRMR = 0.055.

The PTSD model indicated significant direct effects for seven of the nine proposed pathways, ranging in strength from *β* = 0.14 to *β* = 0.69. Overall, the pattern of effects was largely consistent with the proposed model. However, attachment insecurity did not have a significant direct effect on PTSD symptoms (*β* = −0.01, *p* = 0.896), instead operating through more proximal mechanisms (e.g., emotion dysregulation). Social connectedness also did not have a significant direct effect on PTSD symptoms (*β* = 0.07, *p* = 0.516). The model explained 35.0% of the variance in PTSD symptoms. Figure 1 presents all direct effects of the model.

A *k =* 5‐fold cross‐validation was conducted to assess the stability of the model. Fit indices were calculated for each fold. The average CFI of 0.948 (SD = 0.003), TLI of 0.925 (SD = 0.004), RMSEA of 0.079 (SD = 0.003) and SRMR of 0.048 (SD = 0.001). These results support the generalisability of the model. Bootstrapped bias‐corrected 95% confidence intervals supported significant indirect pathways to PTSD through emotion regulation, including sequential pathways through emotion identification and emotion regulation, whereas indirect pathways through social connectedness were not significant. Table 3 shows these results.

**TABLE 3:** Standardised parameter estimates for indirect and total effects for PTSD.

Path	*β*	SE	95% CI (lower)	95% CI (upper)	*p*
Indirect effects					
Attachment > ER > PTSD	0.12	0.02	0.05	0.13	< 0.001
Attachment > EI > ER > PTSD	0.20	0.03	0.09	0.20	< 0.001
Attachment > social connectedness > PTSD	0.04	0.05	−0.07	0.13	0.516
ACEs > attachment > ER > PTSD	0.03	0.01	0.01	0.04	< 0.001
ACEs > attachment > EI > ER > PTSD	0.06	0.01	0.02	0.06	< 0.001
ACEs > attachment > social connectedness > PTSD	0.01	0.02	−0.02	0.04	0.517
Total effects					
ACEs on PTSD	0.24	0.03	0.12	0.24	< 0.001
Attachment on PTSD	0.35	0.03	0.20	0.32	< 0.001
ER on PTSD	0.51	0.07	0.28	0.54	< 0.001
EI on PTSD	−0.35	0.05	−0.41	−0.21	< 0.001
Social connectedness on PTSD	0.07	0.10	−0.13	0.27	0.516

*Note:* The total variance explained by the model (*R* ^2^) was 35.0%.

Abbreviations: ACES, adverse childhood experiences; EI, emotion identification; ER, emotion regulation; PTSD, post‐traumatic stress disorder.

#### 3.4.2 Submodels of PTSD With Attachment Anxiety and Attachment Avoidance

The model was replicated using attachment anxiety and attachment avoidance separately; direct effects of each model are displayed in Figures S1 and S2 and indirect and total effects are shown in Tables S2 and S3, respectively. When using attachment anxiety, results closely mirrored the main PTSD model. When using attachment anxiety, results closely mirrored the main PTSD model, with the significance and direction of all direct effects remaining unchanged. The model explained 34.6% of the variance in ICD‐11 PTSD symptoms.

When using attachment avoidance, one difference emerged: attachment avoidance had no significant direct effect on emotional dysregulation (*β* = 0.03, *p* = 0.439), rendering the indirect path through emotion regulation non‐significant. However, the total effect of attachment avoidance on PTSD was higher than that of attachment anxiety, operating instead through other mediators (i.e., emotion identification and emotion regulation). This model explained 34.2% of the variance in PTSD symptoms.

#### 3.4.3 High‐Symptom Feasibility Analysis for PTSD Outcomes

The PTSD model was re‐estimated among participants meeting the ICD‐11 symptom‐cluster thresholds for either PTSD or CPTSD. The model demonstrated acceptable‐to‐good fit, *χ* ^2^(44) = 90.59, *p* < 0.001, CFI = 0.955, TLI = 0.932, RMSEA = 0.067 and SRMR = 0.058. The pattern of direct associations was consistent with the full‐sample model. The model explained 35.2% of the variance in PTSD symptoms. Direct, indirect and total effects are reported in Table S4.

#### 3.4.4 Complex Post‐Traumatic Stress Symptoms

After the measurement model of CPTSD conducted, indicating generally good fit, SEM path model was implemented. The SEM model for CPTSD symptoms demonstrated a good fit: *χ* ^2^(34) = 145.63, *p* < 0.001, CFI = 0.976, TLI = 0.962, RMSEA = 0.068 and SRMR = 0.056.

The structural model for *CPTSD* mirrored the PTSD model in overall structure and pattern of effects. The range of *β* strength sizes for significant effects was between 0.12 to 0.70 (overall range: −0.70 to 0.57); however, one non‐significant association was the relationship between attachment insecurity and CPTSD symptoms (*β =* −0.05, *p* = 0.158). Overall, the results generally aligned with the hypothesised CPTSD model. However, similar to the PTSD model, the non‐significant direct effect of attachment insecurity indicates that its influence on CPTSD symptoms operates indirectly through socio‐affective mediators. Direct effects of the SEM model for CPTSD symptoms are indicated in Figure 2.

The SEM model was evaluated using *k* = 5‐fold cross‐validation. The mean and standard deviation of these indices were calculated. Across the folds, the model demonstrated good fit, with an average CFI of 0.967 (SD = 0.01), TLI of 0.946 (SD = 0.01), RMSEA of 0.081 (SD = 0.01) and SRMR of 0.065 (SD = 0.01). These results support the model's generalisability. Bootstrapped bias‐corrected 95% confidence intervals demonstrated all indirect and total pathways were significant. See Table 4 for results.

**TABLE 4:** Standardised parameter estimates for indirect and total effects for CPTSD.

Path	*β*	SE	95% CI (lower)	95% CI (upper)	*p*
Indirect effects					
Attachment > ER > CPTSD	0.14	0.02	0.11	0.21	< 0.001
Attachment > EI > ER > CPTSD	0.22	0.02	0.18	0.26	< 0.001
Attachment > social connectedness > CPTSD	0.25	0.03	0.19	0.31	< 0.001
ACES > attachment > ER > CPTSD	0.04	0.01	0.03	0.06	< 0.001
ACES > attachment > EI > ER > CPTSD	0.06	0.01	0.04	0.08	< 0.001
ACES > attachment > social connectedness > CPTSD	0.07	0.01	0.04	0.09	< 0.001
Total effects					
ACES on CPTSD	0.29	0.03	0.23	0.35	< 0.001
Attachment on CPTSD	0.55	0.03	0.5	0.6	< 0.001
ER on CPTSD	0.57	0.04	0.62	0.72	< 0.001
EI on CPTSD	−0.4	0.03	−0.46	−0.34	< 0.001
Social connectedness on CPTSD	0.36	0.06	0.23	0.48	< 0.001

*Note:* The total variance explained by the model (*R* ^2^): 75.7%.

Abbreviations: ACEs, adverse childhood experiences; CPTSD, complex post‐traumatic stress disorder; EI, emotion identification; ER, emotion regulation.

#### 3.4.5 Submodels of CPTSD With Attachment Anxiety and Attachment Avoidance

The model was conducted using attachment anxiety and attachment avoidance separately, direct effects of each model are displayed in Figures S3 and S4 and total and indirect effects shown in Tables S5 and S6, respectively. When attachment anxiety was the sole attachment dimension used, the results were consistent with the main CPTSD symptom model. Significance and direction of effect sizes directly emulated the main model, and the total variance explained in CPTSD symptoms was 74.4%.

When attachment avoidance was implemented as the only attachment dimension, one key difference emerged: the direct effect of attachment on emotion dysregulation was not statistically significant (*β* = 0.02, *p* = 0.430). This replicates the findings of the submodel of PTSD using attachment avoidance above, indicating attachment avoidance buffers the experience of emotional dysregulation The model explained 75.4% of the total variance in CPTSD symptoms.

#### 3.4.6 Sensitivity Analysis Controlling for Trauma Repetition

The CPTSD model was also re‐estimated controlling for trauma repetition, which was allowed to covary with ACEs. The adjusted model demonstrated good fit, *χ* ^2^(43) = 177.06, *p* < 0.001, CFI = 0.972, TLI = 0.957, RMSEA = 0.067 and SRMR = 0.068. Trauma repetition showed a small independent association with CPTSD symptoms (*β* = 0.06, *p* = 0.012), while the primary structural coefficients remained largely unchanged. Explained variance increased only slightly, from 75.7% to 76.8%.

#### 3.4.7 High‐Symptom Feasibility Analysis for CPTSD Outcomes

The CPTSD model was re‐estimated among participants meeting the ICD‐11 symptom‐cluster thresholds for either PTSD or CPTSD. The model demonstrated good fit, *χ* ^2^(34) = 67.72, *p* = 0.001, CFI = 0.972, TLI = 0.954, RMSEA = 0.064, 90% CI [0.042, 0.087] and SRMR = 0.055. The pattern of direct associations was consistent with the full‐sample model, with all pathways retaining the same direction and statistical significance. The model explained 71.7% of the variance in CPTSD symptoms. Direct, indirect and total effects are reported in Table S7.

### 3.5 Attachment Across ICD‐11 PTSD and DSO Symptom Clusters

To examine whether attachment showed differential associations across ICD‐11 PTSD and DSO symptom clusters, supplementary Pearson correlations were conducted between attachment insecurity and the six symptom clusters (Table 8). Total attachment insecurity was significantly associated with all symptom clusters (Pearson's *r* = 0.23–0.55, all FDR‐adjusted *p* < 0.001), with the strongest association observed for disturbances in relationships (*r* = 0.55). Attachment anxiety was most strongly associated with affective dysregulation (*r* = 0.40), whereas attachment avoidance was most strongly associated with disturbances in relationships (*r* = 0.49).

## 4 Discussion

The present study introduces and evaluates the intra‐interpersonal vulnerability model of post‐traumatic stress symptoms. The model was produced from developmental and attachment theory, social processes and dominant theories in emotion regulation and, as such, represents a social‐affective developmental model of post‐traumatic stress. The results showed excellent fit and generalisability. The model showed broadly comparable patterns of associations across ICD‐11 PTSD and CPTSD, particularly for the developmental and emotion‐related pathways. Attachment insecurity was not directly associated with either PTSD or CPTSD symptoms, instead operating indirectly through more proximal mechanisms. Emotion dysregulation was associated with both PTSD and CPTSD symptoms, whereas social connectedness was associated with CPTSD but not PTSD symptoms. Overall, our intra‐interpersonal vulnerability model conceptualises trauma vulnerability as emerging from insecure relational patterns that progressively disrupt emotional clarity, regulation and social connection. The model showed broadly comparable patterns of associations for both PTSD and CPTSD.

Hypothesis 1 was supported, which is that ACEs would impact trauma‐related outcomes both directly and indirectly through their influence on attachment insecurity and emotion regulation. While this pattern is well‐established in the literature, it reinforces prior findings (e.g., Crow et al. 2021), that attachment and regulatory difficulties jointly explain the lasting impact of early adversity, even when controlling for maltreatment. In other words, childhood trauma not only contributes to post‐traumatic stress but also fosters attachment insecurity, which shapes long‐term psychological vulnerability (Cloitre et al. 2019; Gondek et al. 2021).

Hypothesis 2 was supported; attachment insecurity had a total effect on PTSD and CPTSD symptoms. However, the direct association between attachment insecurity and both PTSD and CPTSD was non‐significant. Instead, it was indirectly linked via mediators. In previous research, there has been considerable heterogeneity in findings related to the relationship between attachment insecurity and trauma symptoms (Woodhouse et al. 2015). Our bivariate correlations aligned closely with these meta‐analytic effect sizes, suggesting that the lack of a direct effect in our model was unlikely to be due to sampling issues. A possible reason for the lack of a direct effect between attachment and PTSD is statistical suppression, as the model included multiple interrelated mediators. With indirect effects operating through emotion identification and regulation across PTSD and CPTSD, and social connectedness for CPTSD, little unique variance may have remained for a direct attachment effect. Exploratory analyses further showed that attachment insecurity was most strongly associated with DSO symptoms. Consistent with this interpretation, developmental attachment accounts propose that disruptions to early attachment relationships can have enduring consequences for both emotion regulation and interpersonal functioning, potentially providing a developmental basis for these broader self‐organisational difficulties (Charuvastra and Cloitre 2008).

Previous research may capture both direct and indirect variance between attachment and PTSD but is largely agnostic to the causal structure linking these associations. The present study extends this work by testing a theoretically specified model of how these associations may arise, in which attachment insecurity is proposed to influence post‐traumatic stress symptoms through more proximal emotional and interpersonal processes. Consistent with attachment theory, our results indicate attachment exerts its influence across time which shapes regulatory capacities, such as emotion regulation (Lim et al. 2020). As a stress‐regulating biological system, attachment influences the development and calibration of key systems such as the HPA axis, oxytocin release and autonomic nervous system regulation (Gunnar and Quevedo 2007)—helping to manage arousal and threat across the lifespan. As such, insecure attachment styles reflect longstanding relational expectations that may limit effective internal regulation, particularly under stress. This dovetails with our finding that ACEs were associated with both attachment and trauma‐related symptoms directly.

Addressing the research question regarding attachment subdimensions, the association between attachment and trauma symptoms appeared more complex when anxiety and avoidance were examined separately. While attachment anxiety, and insecure attachment overall, was associated with emotion regulation difficulties, attachment avoidance showed no significant association with emotion dysregulation. This echoes results from Tammilehto et al. (2022), who, using ecological momentary assessment, found that trait attachment avoidance did not predict any attempts at emotion regulation. The authors suggest this was likely due to avoidantly attached individuals struggling to monitor and articulate their daily emotional experiences. Our results support this highlighting how attachment avoidance is linked to reduced capacity for identifying emotions, in turn, impacting emotion regulation and trauma‐related symptoms. Relatedly, the ability to identify emotions is diminished in several psychological disorders (e.g., panic disorder, borderline personality disorder and major depressive disorder)—recognising its importance within the transdiagnostic process.

Hypothesis 3 was supported: emotion identification significantly mediated the relationship between attachment insecurity and emotion regulation difficulties, which in turn predicted all trauma‐related outcomes. The findings related to emotion regulation provide strong empirical support for role of emotion identification as a foundational component in regulating distress. This sequential pattern reflects the theoretical premise that individuals must first accurately identify and understand their emotional states before they can effectively select or implement regulatory strategies (Sheppes et al. 2015). Moreover, beliefs about the controllability of emotion—often shaped by early attachment and later reinforced through dysregulated experiences—may further determine the strategies individuals choose to employ (Kneeland et al. 2016). Those who perceive emotions as uncontrollable may be more likely to disengage or suppress emotional responses, thereby reinforcing distress. Together, these findings reinforce the value of distinguishing emotion clarity from regulation and support targeting these mechanisms in trauma‐focused interventions.

Hypothesis 4 was partially supported. Social connectedness significantly mediated the relationship between attachment insecurity and CPTSD symptoms, whereas the corresponding indirect pathway for ICD‐11 PTSD was small and non‐significant. This does not suggest that social connectedness is unrelated to PTSD; substantial evidence demonstrates associations between social support and PTSD, including small but robust prospective effects (Wang et al. 2021). Seeking social networks in the wake of mass trauma is a common coping strategy and delays in forming these connections are linked to an increased risk of developing psychopathology (Bryant 2023). Results are consistent with the notion that individuals with secure attachment often anticipate care and support from others to alleviate distress. In contrast, those with insecure attachment may not expect support, limiting opportunities to regulate their distress (Mikulincer et al. 2015). The present findings extend this perspective by suggesting that social connectedness may not contribute uniformly across post‐traumatic stress presentations. Specifically, this interpersonal pathway may be more salient for CPTSD, where relational disturbance forms part of the DSO symptom profile, while its contribution to the core symptoms of ICD‐11 PTSD may be comparatively limited.

### 4.1 Limitations and Future Directions

One limitation of this study is that it was conducted using a community sample rather than a clinical population. This may limit the generalisability of the findings to more severe or clinically diagnosed cases, as individuals in a community sample may not show the same levels of symptom severity or impairment as those seeking clinical treatment. However, based on diagnostic screening measures, a substantial proportion of the sample met criteria for probable ICD‐11 PTSD or CPTSD. Future research could include both community and clinical samples to better understand how these factors operate across different levels of symptomatology. Another limitation relating to the sample was that gender distribution was skewed towards female participants, which may constrain generalisability. Future studies should aim to replicate these findings in more gender‐balanced samples.

This study used cross‐sectional data, which restricts the ability to determine temporal sequences and the developmental progression this study proposes. Other research methods can be utilised to better understand these relationships; for instance, retrospective reporting and longitudinal research may assist in exploring how specific attachment and socio‐affective variables contribute to trauma symptoms.

The self‐report measures used in this study are common and well‐validated but can be limited when assessing underlying processes for certain constructs. For instance, self‐report measures for assessing emotion identification deficits can be problematic as these individuals may lack objective insight. Future research could consider multimodal assessments, including clinician‐administered interviews such as the Toronto Structured Interview for Alexithymia (Bagby et al. 2006) or psychophysiological measures. This may assist in testing mechanistic pathways and causal hypotheses.

### 4.2 Clinical Implication

This study highlights the importance of targeting key intrapersonal and interpersonal processes—rather than attachment itself—in treating PTSD and CPTSD. This does not undermine the significance of attachment as a developmental risk factor for post‐traumatic stress; as such, attachment may act as a pertinent, yet distal, factor by shaping downstream processes such as emotion regulation and social functioning. Indeed, there is limited evidence to suggest attachment‐oriented therapies resolve post‐traumatic stress conditions. A small trial of trauma‐focused CBT found that while attachment improved during treatment, baseline attachment did not predict PTSD outcomes (Allen and Brown 2023), suggesting that attachment may exert its influence on PTSD indirectly. This may be due to attachment representing a more stable disposition, whereas abilities such as emotional awareness and the capacity to select and apply regulatory strategies may provide more proximal and modifiable treatment targets. Treatment may therefore be informed by identifying individual difficulties, such as limited emotional clarity, difficulties selecting and implementing regulatory strategies or reduced capacity to access and maintain supportive relationships.

Rather than supporting a single therapeutic modality, the present findings suggest that treatment may benefit from formulating the specific affective and interpersonal processes contributing to an individual's difficulties and selecting interventions accordingly. Several existing approaches to complex trauma explicitly target these domains (Ford and Courtois 2020). For example, Enhanced STAIR (ESTAIR) was developed as a flexible modular treatment for CPTSD, comprising modules addressing emotion regulation, relationship patterns, self‐concept and trauma processing (Karatzias et al. 2023). This modular approach is consistent with the present findings, as treatment components can be selected and sequenced according to the specific affective and interpersonal difficulties experienced by the individual.

## 5 Conclusion

This study tested a newly proposed intra‐interpersonal vulnerability model of post‐traumatic stress that demonstrated equivalent structure and pathways across ICD‐11 PTSD and CPTSD. Results highlight emotion regulation as a proximal factor across PTSD and CPTSD, with social connectedness emerging as an additional interpersonal pathway for CPTSD. These protective and risk factors are, in turn, largely influenced by disrupted attachment security, echoing with research highlighting the impact of attachment on the biological stress system. As such, insecure attachment primes an individual to develop severe post‐traumatic stress symptoms, as they often struggle to feel supported by others and have difficulty making sense of their internal affective states and, in turn, face challenges in self‐regulation. These findings support tailoring interventions to individual emotional and relational needs in trauma treatment.

## Conflicts of Interest

The authors declare no conflicts of interest.

## Supporting information


**Table S1:** Gender differences across primary study variables.
**Table S2:** Standardised parameter estimates for indirect and total effects for PTSD using attachment anxiety.
**Table S3:** Standardised parameter estimates for indirect and total effects for PTSD using attachment avoidance.
**Table S4:** Direct, indirect and total effects for the ICD‐11 PTSD model among participants meeting PTSD or CPTSD symptom‐cluster thresholds.
**Table S5:** Standardised parameter estimates for indirect and total effects for complex PTSD using attachment anxiety.
**Table S6:** Standardised parameter estimates for indirect and total effects for complex PTSD using attachment avoidance.
**Table S7:** Direct, indirect and total effects for the ICD‐11 complex PTSD model among participants meeting PTSD or CPTSD symptom‐cluster thresholds.
**Table S8:** Pearson correlations between attachment and ICD‐11 PTSD and CPTSD symptom clusters.
**Figure S1:** Structural equation model for ICD‐11 PTSD with attachment anxiety.
**Figure S2:** Structural equation model for ICD‐11 PTSD with attachment avoidance.
**Figure S3:** Structural equation model for CPTSD with attachment anxiety.
**Figure S4:** Structural equation model for CPTSD with attachment avoidance.

## Data Availability

The data that support the findings of this study are available from the corresponding author upon reasonable request. Data are not publicly available due to ethical and privacy restrictions relating to trauma‐related participant information.
