# Supplementary material for: An Intra‐Interpersonal Vulnerability Model for Understanding Trauma Conditions: Factors in the Development and Maintenance of PTSD and Complex PTSD
Source: Clin Psychol Psychother. 2026 Sep 1;33(5):e70333. doi: 10.1002/cpp.70333 (PMC13533850; doi:10.1002/cpp.70333)
Supplement: Supplementary file 1 [file CPP-33-e70333-s001.docx]

**Supplementary materials**

**Supplementary Table 1.**

***Gender Differences Across Primary Study Variables***

| **Variable** | **Male M (SD)** | **Female M (SD)** | **t** | **p** | **FDR p** | **Cohen’s d** |
| --- | --- | --- | --- | --- | --- | --- |
| Adverse childhood experiences | 4.08 (3.22) | 5.02 (3.30) | −3.01 | .003 | **.016** | −.29 |
| Attachment insecurity | 36.89 (11.59) | 37.81 (12.17) | −0.80 | .422 | .469 | −.08 |
| Emotion regulation difficulties | 82.99 (22.97) | 89.66 (23.53) | −2.98 | .003 | **.016** | −.28 |
| Emotional identification | 34.58 (9.23) | 33.94 (9.32) | 0.72 | .471 | .471 | .07 |
| Difficulty identifying feelings | 18.65 (7.15) | 19.64 (7.30) | −1.42 | .157 | .235 | −.14 |
| Personal beliefs | 13.77 (3.82) | 13.27 (3.42) | 1.40 | .164 | .235 | .14 |
| Emotional loneliness | 18.56 (5.62) | 18.08 (5.83) | 0.87 | .387 | .469 | .08 |
| Perceived social support | 55.01 (17.28) | 57.76 (16.16) | −1.66 | .099 | .235 | −.17 |
| ICD-11 PTSD/CPTSD symptoms | 19.02 (11.44) | 21.35 (11.42) | −2.10 | .037 | .125 | −.20 |
| Note. Gender comparisons included participants identifying as male (n = 132) or female (n = 542); 33 participants with other or missing gender responses were excluded from these analyses. Gender differences were examined using Welch’s independent-samples t-tests. P-values were adjusted using the Benjamini–Hochberg false discovery rate (FDR) procedure. Cohen’s d represents the standardised mean difference, with negative values indicating higher scores among females. Boldface indicates statistically significant FDR-adjusted p-values. ICD-11 = International Classification of Diseases, 11th Revision; PTSD = post-traumatic stress disorder; CPTSD = complex post-traumatic stress disorder. | | | | | | |

**Supplementary Table 2.**

*Standardised Parameter Estimates for Indirect and Total Effects for PTSD using Attachment Anxiety*

| **Path** | ***B*** | ***SE*** | **95% CI for *B*** | ***β*** | ***p*** |
| --- | --- | --- | --- | --- | --- |
| **Indirect effects** |  |  |  |  |  |
| Attachment anxiety → ER → PTSD | .103 | .019 | [.066, .140] | .140 | < .001 |
| Attachment anxiety → EI → ER → PTSD | .103 | .019 | [.066, .140] | .140 | < .001 |
| Attachment anxiety → Social Connectedness → PTSD | .025 | .028 | [−.031, .081] | .034 | .384 |
| ACEs → Attachment anxiety → PTSD | −.014 | .009 | [−.031, .003] | −.019 | .107 |
| ACEs → Attachment anxiety → ER → PTSD | .026 | .006 | [.014, .038] | .035 | < .001 |
| ACEs → Attachment anxiety → EI → ER → PTSD | .026 | .006 | [.014, .038] | .035 | < .001 |
| ACEs → Attachment anxiety → Social Connectedness → PTSD | .006 | .007 | [−.008, .020] | .008 | .387 |
| **Total effects** |  |  |  |  |  |
| ACEs on PTSD | .151 | .028 | [.096, .206] | .205 | < .001 |
| Attachment anxiety on PTSD | .174 | .031 | [.113, .235] | .236 | < .001 |
| ER on PTSD | .440 | .064 | [.315, .565] | .539 | < .001 |
| EI on PTSD | −.351 | .052 | [−.453, −.248] | −.398 | < .001 |
| Social Connectedness on PTSD | .064 | .073 | [−.079, .206] | .069 | .383 |
| **Note.** B = unstandardised estimate; SE = standard error; CI = confidence interval; β = fully standardised estimate. ACEs = adverse childhood experiences; EI = emotion identification; ER = emotion regulation; PTSD = post-traumatic stress disorder. The model explained **34.6% of the variance in ICD-11 PTSD symptoms (**R**² = .346).** | | | | | |

**Supplementary Table 3.**

*Standardised Parameter Estimates for Indirect and Total Effects for PTSD using Attachment Avoidance*

| **Path** | **B** | **SE** | **95% CI for B** | **β** | **p** |
| --- | --- | --- | --- | --- | --- |
| **Indirect effects** |  |  |  |  |  |
| Attachment avoidance → ER → PTSD | .010 | .013 | [−.015, .035] | .013 | .444 |
| Attachment avoidance → EI → ER → PTSD | .147 | .027 | [.095, .200] | .201 | < .001 |
| Attachment avoidance → Social Connectedness → PTSD | .004 | .044 | [−.081, .090] | .006 | .923 |
| ACEs → Attachment avoidance → PTSD | .009 | .007 | [−.005, .023] | .012 | .198 |
| ACEs → Attachment avoidance → ER → PTSD | .002 | .002 | [−.003, .006] | .002 | .449 |
| ACEs → Attachment avoidance → EI → ER → PTSD | .026 | .007 | [.012, .041] | .036 | < .001 |
| ACEs → Attachment avoidance → Social Connectedness → PTSD | .001 | .008 | [−.015, .016] | .001 | .923 |
| **Total effects** |  |  |  |  |  |
| ACEs on PTSD | .144 | .028 | [.088, .199] | .196 | < .001 |
| Attachment avoidance on PTSD | .212 | .031 | [.153, .272] | .289 | < .001 |
| ER on PTSD | .415 | .067 | [.283, .547] | .513 | < .001 |
| EI on PTSD | −.364 | .061 | [−.483, −.245] | −.417 | < .001 |
| Social Connectedness on PTSD | .011 | .111 | [−.207, .228] | .010 | .923 |
| **Note.** B = unstandardised estimate; SE = standard error; CI = confidence interval; β = fully standardised estimate. ACEs = adverse childhood experiences; EI = emotion identification; ER = emotion regulation; PTSD = post-traumatic stress disorder. The model explained **34.2% of the variance in ICD-11 PTSD symptoms (**R**² = .342).** | | | | | |

**Supplementary Table 4.**

*Direct, Indirect, and Total Effects for the ICD-11 PTSD Model Among Participants Meeting PTSD or CPTSD Symptom-Cluster Thresholds*

| **Path** | **B** | **SE** | **95% CI for B** | **β** | **p** |
| --- | --- | --- | --- | --- | --- |
| **Direct effects** |  |  |  |  |  |
| Emotion dysregulation → PTSD | — | — | — | .497 | .002 |
| Social connectedness → PTSD | — | — | — | .181 | .290 |
| ACEs → PTSD | — | — | — | .228 | .008 |
| Attachment insecurity → PTSD | — | — | — | −.201 | .079 |
| Attachment insecurity → emotion dysregulation | — | — | — | .242 | < .001 |
| Emotion identification → emotion dysregulation | — | — | — | −.643 | < .001 |
| Attachment insecurity → emotion identification | — | — | — | −.434 | < .001 |
| ACEs → attachment insecurity | — | — | — | .242 | < .001 |
| Attachment insecurity → social connectedness | — | — | — | .570 | < .001 |
| **Indirect effects** |  |  |  |  |  |
| Attachment → ER → PTSD | .043 | .018 | [.008, .077] | .120 | .015 |
| Attachment → EI → ER → PTSD | .049 | .018 | [.015, .083] | .138 | .005 |
| Attachment → Social → PTSD | .037 | .035 | [−.031, .105] | .103 | .292 |
| ACEs → Attachment → ER → PTSD | .010 | .005 | [.000, .020] | .029 | .040 |
| ACEs → Attachment → EI → ER → PTSD | .012 | .005 | [.002, .022] | .033 | .024 |
| ACEs → Attachment → Social → PTSD | .009 | .009 | [−.008, .026] | .025 | .309 |
| **Total effects** |  |  |  |  |  |
| ACEs → PTSD | .111 | .035 | [.043, .179] | .315 | .001 |
| Attachment → PTSD | .057 | .031 | [−.003, .117] | .160 | .064 |
| ER → PTSD | .205 | .066 | [.076, .334] | .497 | .002 |
| EI → PTSD | −.135 | .045 | [−.223, −.047] | −.319 | .003 |
| Social → PTSD | .097 | .091 | [−.082, .276] | .181 | .290 |
| **Note.** B = unstandardized estimate; SE = standard error; CI = confidence interval; β = standardized estimate; ACEs = adverse childhood experiences; EI = emotion identification; ER = emotion regulation; PTSD = posttraumatic stress disorder. The model explained 35.2% of the variance in PTSD symptoms (R² = .352). | | | | | |

**Supplementary Table 5.**

*Standardised Parameter Estimates for Indirect and Total Effects for Complex PTSD using Attachment Anxiety*

| Path | *β* | SE | 95% CI (Lower) | 95% CI (Upper) | *p*-value |
| --- | --- | --- | --- | --- | --- |
| *Indirect effects* | | | | | |
| Attachment -> ER > CPTSD | 0.16 | 0.02 | 0.11 | 0.20 | < .001 |
| Attachment > EI > ER > CPTSD | 0.16 | 0.02 | 0.12 | 0.20 | < .001 |
| Attachment > Social connectedness > CPTSD | 0.15 | 0.03 | 0.09 | 0.21 | < .001 |
| ACES > Attachment > ER > CPTSD | 0.04 | 0.01 | 0.02 | 0.06 | < .001 |
| ACES >Attachment > EI > ER > CPTSD | 0.04 | 0.01 | 0.02 | 0.05 | < .001 |
| ACES >Attachment > Social connected > CPTSD | 0.04 | 0.01 | 0.02 | 0.06 | < .001 |
| *Total effects* | | | | | |
| ACES on CPTSD | 0.24 | 0.03 | 0.19 | 0.30 | < .001 |
| Attachment on CPTSD | 0.43 | 0.03 | 0.37 | 0.49 | < .001 |
| ER on CPTSD | 0.60 | 0.05 | 0.51 | 0.70 | < .001 |
| EI on CPTSD | -0.44 | 0.04 | -0.52 | -0.37 | < .001 |
| Social connectedness on CPTSD | 0.31 | 0.05 | 0.21 | 0.41 | < .001 |
| **Note:** ACES, Adverse Childhoold Experiences; CPTSD, Complex Post-Traumatic Stress Disorder; EI, Emotion Identification; ER, Emotion Regulation. The total variance explained by the model (R²): 74.4% | | | | | |

**Supplementary Table 6.**

*Standardised Parameter Estimates for Indirect and Total Effects for Complex PTSD using Attachment Avoidance*

| Path | *β* | SE | 95% CI (Lower) | 95% CI (Upper) | *p*-value |
| --- | --- | --- | --- | --- | --- |
| *Indirect effects* | | | | | |
| Attachment -> ER > CPTSD | 0.02 | 0.02 | -0.02 | 0.05 | 0.433 |
| Attachment > EI > ER > CPTSD | 0.22 | 0.03 | 0.17 | 0.28 | < .001 |
| Attachment > Social connectedness > CPTSD | 0.20 | 0.04 | 0.12 | 0.29 | < .001 |
| ACES > Attachment > ER > CPTSD | 0.00 | 0.00 | 0.00 | 0.01 | 0.439 |
| ACES >Attachment > EI > ER > CPTSD | 0.04 | 0.01 | 0.02 | 0.06 | < .001 |
| ACES >Attachment > Social connected > CPTSD | 0.04 | 0.01 | 0.02 | 0.06 | .001 |
| *Total effects* | | | | | |
| ACES on CPTSD | 0.20 | 0.03 | 0.15 | 0.26 | < .001 |
| Attachment on CPTSD | 0.39 | 0.03 | 0.33 | 0.46 | < .001 |
| ER on CPTSD | 0.57 | 0.05 | 0.46 | 0.67 | < .001 |
| EI on CPTSD | -0.46 | 0.05 | -0.56 | -0.37 | < .001 |
| Social connectedness on CPTSD | 0.36 | 0.07 | 0.23 | 0.49 | < .001 |
| **Note:** ACES, Adverse Childhoold Experiences; CPTSD, Complex Post-Traumatic Stress Disorder; EI, Emotion Identification; ER, Emotion Regulation. The total variance explained by the model (R²): 75.4% | | | | | |

**Supplementary Table 7.**

*Direct, Indirect, and Total Effects for the ICD-11 Complex PTSD Model Among Participants Meeting PTSD or CPTSD Symptom-Cluster Thresholds*

| **Path** | **B** | **SE** | **95% CI for B** | **β** | **p** |
| --- | --- | --- | --- | --- | --- |
| **Direct effects** |  |  |  |  |  |
| Emotion Regulation → CPTSD | — | — | — | .614 | < .001 |
| Social Connectedness → CPTSD | — | — | — | .306 | .014 |
| ACEs → CPTSD | — | — | — | .113 | .010 |
| Attachment → CPTSD | — | — | — | −.021 | .750 |
| Attachment → Emotion Regulation | — | — | — | .245 | < .001 |
| Emotion Identification → Emotion Regulation | — | — | — | −.646 | < .001 |
| Attachment → Emotion Identification | — | — | — | −.434 | < .001 |
| ACEs → Attachment | — | — | — | .242 | < .001 |
| Attachment → Social Connectedness | — | — | — | .568 | < .001 |
| **Indirect effects** |  |  |  |  |  |
| Attachment → ER → CPTSD | .046 | .015 | [.016, .076] | .150 | .003 |
| Attachment → EI → ER → CPTSD | .053 | .014 | [.026, .079] | .172 | < .001 |
| Attachment → Social Connectedness → CPTSD | .053 | .022 | [.010, .096] | .174 | .017 |
| ACEs → Attachment → ER → CPTSD | .011 | .005 | [.002, .020] | .036 | .019 |
| ACEs → Attachment → EI → ER → CPTSD | .013 | .005 | [.004, .022] | .042 | .007 |
| ACEs → Attachment → Social Connectedness → CPTSD | .013 | .006 | [.000, .025] | .042 | .042 |
| **Total effects** |  |  |  |  |  |
| ACEs on CPTSD | .071 | .020 | [.032, .109] | .233 | < .001 |
| Attachment on CPTSD | .145 | .028 | [.090, .200] | .475 | < .001 |
| Emotion Regulation on CPTSD | .222 | .046 | [.132, .312] | .614 | < .001 |
| Emotion Identification on CPTSD | −.145 | .032 | [−.207, −.082] | −.396 | < .001 |
| Social Connectedness on CPTSD | .140 | .057 | [.029, .251] | .306 | .014 |
| **Note.** ACEs = adverse childhood experiences; CPTSD = complex post-traumatic stress disorder; EI = emotion identification; ER = emotion regulation. B = unstandardised estimate; SE = standard error; CI = confidence interval; β = fully standardised estimate. Dashes indicate that unstandardised estimates and confidence intervals were not reported for direct effects in this sensitivity analysis. The model explained 71.7% of the variance in CPTSD symptoms (R² = .717). | | | | | |

**Supplementary Table 8**
Pearson Correlations Between Attachment and ICD-11 PTSD and CPTSD Symptom Clusters

| **Attachment variable** | **Re-experiencing** | **Avoidance** | **Sense of Threat** | **Affective Dysregulation** | **Negative Self-Concept** | **Disturbances in Relationships** |
| --- | --- | --- | --- | --- | --- | --- |
| Total attachment insecurity | .23 | .32 | .34 | .48 | .44 | **.55** |
| Attachment anxiety | .16 | .22 | .24 | **.40** | .32 | .33 |
| Attachment avoidance | .17 | .26 | .27 | .32 | .34 | **.49** |
| Note. Values are Pearson’s r. All correlations remained significant following Benjamini–Hochberg false discovery rate correction (all adjusted ps < .001). | | | | | | |

**Supplementary Figure 1.** Structural Equation Model for ICD-11 PTSD with Attachment Anxiety

**Supplementary Figure 2.** Structural Equation Model for ICD-11 PTSD with Attachment Avoidance

**Supplementary Figure 3.** Structural Equation Model for CPTSD with Attachment Anxiety

**Supplementary Figure 4.** Structural Equation Model for CPTSD with Attachment Avoidance
